# Newborn screening and prophylactic interventions for sickle cell disease in 47 countries in sub-Saharan Africa: a cost-effectiveness analysis

**DOI:** 10.1186/s12913-016-1572-6

**Published:** 2016-07-26

**Authors:** Andreas Kuznik, Abdulrazaq G. Habib, Deogratias Munube, Mohammed Lamorde

**Affiliations:** 1 Infectious Diseases Institute, Makerere University College of Health Sciences, Kampala, Uganda; 2 Global Health Economics and Outcomes Research, Regeneron Pharmaceuticals, Tarrytown, NY USA; 3 Infectious and Tropical Diseases Unit, Bayero University, Kano, Nigeria; 4 Department of Paediatrics and Child Health, Makerere University College of Health Sciences, Kampala, Uganda

**Keywords:** Anaemia, Sickle cell, Cost-effectiveness analysis, Neonatal screening, Africa

## Abstract

**Background:**

Sickle cell disease (SCD) constitutes a major public health problem in sub-Saharan Africa (SSA). Newborn screening and early subsequent clinical intervention can reduce early mortality and increase life expectancy, but have not been widely implemented in SSA. This analysis assesses the cost-effectiveness of a newborn screening and prophylactic intervention (NSPI) package for SCD in 47 SSA countries.

**Methods:**

A lifetime Markov model with annual cycles was built with infants either being screened using isoelectric focusing (IEF) or not screened. Confirmed positive cases received interventions including insecticide-treated mosquito bed nets, folic acid supplementation, prophylactic antimalarial and penicillin therapy, and vaccinations against bacterial infections. Estimates for the local incidence of SCD, the life expectancy of untreated children, the SCD disability weight, and the cost of screening laboratory tests were based on published sources. Among treated infants, the annual probability of mortality until 30 years of age was derived from a pediatric hospital-based cohort. The outcome of interest included a country-specific cost per Disability Adjusted Life Year (DALY) averted.

**Results:**

Of 47 modeled countries in SSA, NSPI is almost certainly highly cost-effective in 24 countries (average cost per DALY averted: US$184); in 10 countries, it is cost-effective in the base case (average cost per DALY averted: US$285), but the results are subject to uncertainty; in the remaining 13, it is most likely not cost-effective. We observe a strong inverse relationship between the incidence rate of SCD and the cost per DALY averted. Newborn screening is estimated to be cost-effective as long as the incidence rate exceeds 0.2–0.3 %, although in some countries NSPI is cost-effective at incidence rates below this range. In total, NSPI could avert over 2.4 million disability adjusted life years (DALYs) annually across SSA.

**Conclusions:**

Using IEF to screen all newborns for SCD plus administration of prophylactic interventions to affected children is cost-effective in the majority of countries in SSA.

## Background

Sickle cell disease (SCD) is a hereditary disorder of hemoglobin (Hb) characterized by inheritance of two abnormal Hb genes, at least one of which is responsible for the production of HbS. The most common clinical phenotype is the homozygote (Hb SS), also known as sickle cell anemia. It is commonly characterized by chronic hemolytic anemia and recurrent vaso-occlusion which is responsible for the painful crises that characterize the disease. In Africa and Asia, SCD occurs in areas where malaria is endemic [1]. Malaria and bacterial infections such as pneumococcal infections result in morbidity and mortality of SCD patients in Sub-Saharan Africa (SSA) [2, 3]. Malaria contributes not only to mortality, but also to anemia and SCD crises [2].

In SSA, SCD is a disease of public health concern. In 2010, the region had nearly 80 % of the projected 306,000 newborns with SCD worldwide [4]. Mortality estimates among SCD children in SSA range from 50–90 % before age 5 [5]. In contrast, life-expectancy for SCD patients has improved in western countries and most patients live to their 40s and 50s [6]. In those countries, newborn screening programs were introduced incorporating testing newborns for SCD plus interventions (penicillin prophylaxis and pneumococcal vaccination) to prevent bacterial infections among diagnosed cases. Although newborn screening was shown to be cost-effective among African American infants [7], this approach has not been widely adopted in SSA. In SSA, malaria is endemic and newborn screening programs require an additional component of malaria prevention. Evidence from prospective studies in Benin and Angola indicate that a package of prophylactic interventions can reduce the infant and under-5 mortality among newborns with SCD to levels similar to those of the general population [8, 9].

Despite the high SCD burden in SSA, newborn screening and prophylactic intervention (NSPI) programs have not been widely implemented due to concerns of affordability, costs, operational challenges and competing priorities. This analysis assesses the cost-effectiveness of a NSPI program in SSA.

## Methods

**Fig. 1:**
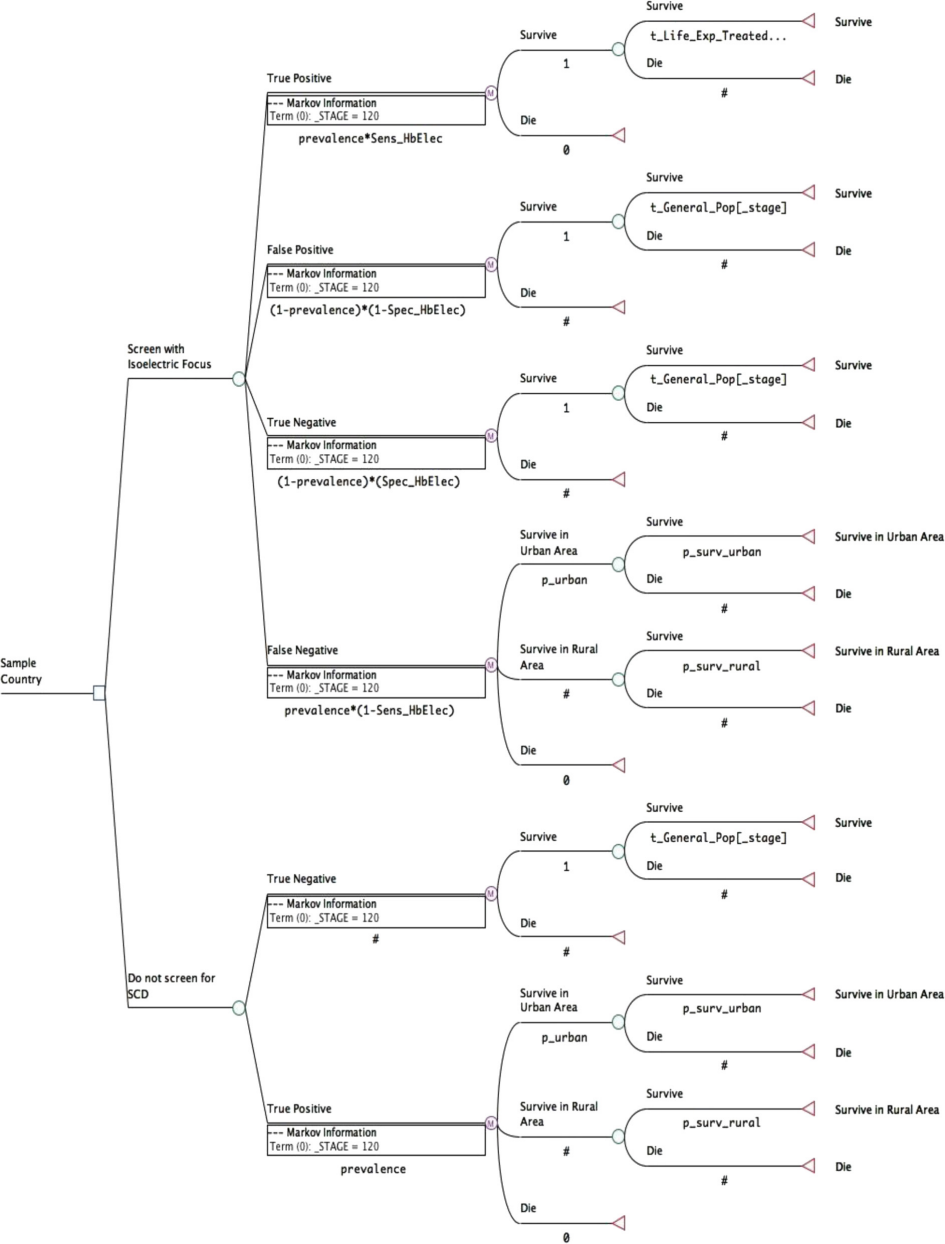
Markov model graphic

A decision analytic model was developed to compare NSPI for SCD relative to no screening, from the perspective of the national health systems in 47 countries in SSA. The model structure was adapted from a previous cost-effectiveness analysis of newborn screening for SCD in the United States [7], and based on a lifetime Markov state transition cohort with annual cycle lengths and the standard half-cycle correction. A graphical representation of the model structure is displayed in Fig. 1. Infants identified to have SCD could either remain alive with SCD, or progress to the terminal steady state of death. In each annual cycle, infants accrued life-years, adjusted for disability. Infants in the NSPI arm of the model consumed health care resources in each cycle. The resource use associated with no screening was assumed to be zero. The common model structure was populated with country-specific model inputs, in order to yield 47 model results.

In the model, every infant was tested at birth for SCD using isoelectric focusing (IEF). Infants with a positive confirmatory test result would initiate an array of prophylactic therapies to reduce the risk of malaria and other infections. Unscreened infants were assumed to remain undiagnosed and untreated. For these infants, the annual probability of mortality was stratified between infants born in rural versus urban areas because prior studies have suggested that infection rates and access to healthcare services could differ between rural and urban areas [5]. A third option of delaying screening upon the onset of symptoms was not included, because SCD-associated mortality may occur before the onset of symptoms, particularly in rural areas. In the intervention arm, the annual probability of mortality among treated infants was stratified for four separate age categories: less than 5 years, 6 years to 20 years, 21 years to 30 years and 31 years and older. For the age categories below 30 years, mortality rates were obtained from a Tanzanian hospital-based cohort and generalized to the other 46 countries included in the model [10]. Beyond 30 years of age, a multiplier was applied to the annual probability of mortality in the general population. All future costs and benefits were discounted at 3 % annually [11]. Calculations were performed using TreeAge Pro 2014, R1.0 (Treeage Software Inc., Williamstown, MA, USA).

Formal ethics approval and consent to participate was not required in this retrospective analysis of observational data.

### Clinical and epidemiologic inputs

General model and country-specific model inputs are displayed in Tables 1 and 2, respectively. The incidence of SCD in newborns was based on published estimates [4]. To generate an estimate of the local incidence rate, the authors reported median annual number of infants born with sickle cell disease for each of the 47 countries of interest and compared these estimates to the annual number of live births in the 2010 United Nations national population estimates [12]. IEF was assumed to have 100 % sensitivity and specificity [13].

**Table 1:** General model inputs

	Basecase	Range		Distribution	Reference
Accuracy of Screening Method					
Sensitivity, Isoelectric Focusing	100.0 %	99.9 %	100.0 %	Exponential	[13]
Specificity, Isoelectric Focusing	100.0 %	99.9 %	100.0 %	Exponential	[13]
Annual Mortality Risk, Untreated					
Rural	37.0 %	13.3 %	64.8 %	Beta	[5]
Urban	13.0 %	4.9 %	24.4 %	Beta	[5]
Annual Mortality Risk, Treated					
<= 5 years	7.3 %	4.1 %	10.5 %	Normal	[10]
6–20 years	1.4 %	1.0 %	1.8 %	Normal	[10]
21–30 years	1.8 %	1.1 %	2.5 %	Normal	[10]
>31 years, general population multiplier	4.5	3.0	6.0	Normal	Own calculation
Disability Weight	0.04	0.02	0.06	Beta	[16]
Costs - Screening					
Isoelectric Focusing – fixed cost per sample	$0.044	$0.022	$0.088	Log-normal	[17]
Isoelectric Focusing – all variable costs	$9.90	$4.45	$18.80	Log-normal	UNHL^a^
Annual costs - treatment					
Pneumoccocal Vaccine, year 1 only	$80.64	$40.32	$120.96	Log-normal	AKTH^b^
Mosquito Net, annual for life	$10.00	$5.00	$15.00	Log-normal	[18]
Proguanil 100 mg, daily for years 1–15	$17.48	$8.74	$26.22	Log-normal	[19]
Proguanil 200 mg, daily for years >15	$34.97	$17.49	$52.46	Log-normal	[19]
Folic Acid 5 mg, daily for life	$0.77	$0.39	$1.16	Log-normal	[19]
Penicillin 125 mg, twice daily for years 1–5	$29.93	$14.97	$44.90	Log-normal	[19]
Penicillin 250 mg, twice daily for years 6–10	$50.37	$25.19	$75.56	Log-normal	[19]
Discount Rate	3.0 %	0.0 %	6.0 %	n/A	[11]

^a^Average cost per isoelectric focusing obtained from Ugandan National Health Laboratory (UNHL) in Kampala, Uganda

^b^Local pharmacy costs obtained from Aminu Kano Teaching Hospital (AKTH), Kano, Nigeria

**Table 2:** Country-specific model inputs

Country name	Live births, 2010 [12]	Median incidence of SCD, 2010 [4]	Incidence rate ([4] divided by [12])	Percentage of population living in rural areas [14]	GDP per capita (US$) 2014 [26]
Angola	758,000	8,364	1.10 %	40.80 %	$5,900
Benin	353,000	4,543	1.29 %	55.10 %	$890
Botswana	45,000	4	0.01 %	38.30 %	$7,240
Burkina Faso	689,000	3,124	0.45 %	73.50 %	$700
Burundi	284,000	889	0.31 %	89.10 %	$270
Cameroon	697,000	6,915	0.99 %	47.90 %	$1,350
Cape Verde	10,000	18	0.18 %	37.40 %	$3,450
Central African Republic	155,000	976	0.63 %	60.90 %	$320
Chad	499,000	2,045	0.41 %	78.20 %	$980
Comoros	25,000	25	0.10 %	72.00 %	$790
Côte d’Ivoire	712,000	3,567	0.50 %	48.70 %	$1,450
Congo-Brazzaville	130,000	1,560	1.20 %	36.30 %	$2,720
DR Congo	2,856,000	38,217	1.34 %	65.70 %	$380
Djibouti	25,000	0	0.00 %	22.90 %	$1,810
Equatorial Guinea	25,000	385	1.54 %	60.50 %	$10,210
Eritrea	179,000	9	0.01 %	78.70 %	$680
Ethiopia	2,550,000	137	0.01 %	83.00 %	$550
Gabon	41,000	864	2.11 %	13.80 %	$9,720
Gambia	65,000	418	0.64 %	42.70 %	$500
Ghana	737,000	5,474	0.74 %	48.10 %	$1,760
Guinea	388,000	5,232	1.35 %	64.60 %	$470
Guinea-Bissau	62,000	192	0.31 %	56.10 %	$550
Kenya	1,507,000	4,475	0.30 %	76.00 %	$1,290
Lesotho	56,000	0	0.00 %	72.40 %	$1,330
Liberia	154,000	562	0.36 %	51.80 %	$370
Madagascar	697,000	3,379	0.48 %	67.40 %	$440
Malawi	698,000	1,688	0.24 %	84.30 %	$250
Mali	601,000	2,701	0.45 %	65.10 %	$650
Mauritania	110,000	433	0.39 %	58.50 %	$1,270
Mauritius	16,000	0	0.00 %	58.20 %	$9,3630
Mozambique	850,000	1,645	0.19 %	68.80 %	$600
Namibia	56,000	30	0.05 %	61.60 %	$5,630
Niger	758,000	4,965	0.66 %	82.20 %	$410
Nigeria	6,219,000	85,186	1.37 %	50.40 %	$2,970
Rwanda	417,000	601	0.14 %	80.90 %	$700
Sao Tome and Principe	5,000	41	0.82 %	37.30 %	$1,670
Senegal	462,000	2,535	0.55 %	57.50 %	$1,050
Sierra Leone	213,000	2,838	1.33 %	60.80 %	$700
Somalia	402,000	13	<0.01 %	62.30 %	$107^30^
South Africa	1,036,000	73	0.01 %	38.00 %	$6,800
Sudan	1,378,000	4,567	0.33 %	66.80 %	$1,710
Swaziland	34,000	4	0.01 %	78.80 %	$3,550
Tanzania	1,846,000	11,022	0.60 %	73.30 %	$920
Togo	210,000	2,175	1.04 %	62.00 %	$570
Uganda	1,484,000	10,143	0.68 %	84.40 %	$670
Zambia	616,000	5,652	0.92 %	60.80 %	$1,680
Zimbabwe	363,000	483	0.13 %	61.40 %	$840
Sum	31,473,000	228,169	0.72 %	N/A	N/A

The under-5 probability of mortality in the unscreened arm of the model was 50 % for infants born in urban areas and 90 % for infants born in rural areas [5]. These under-5 mortality rates were converted into annual mortality rates of 13 % in urban and 37 % in rural areas, respectively, and carried forward in all future cycles beyond the age of 5. The country-specific proportions of rural and urban populations was obtained from a 2011 United Nations report [14]. For screened and treated infants, the observed annual probability of mortality from the Tanzanian cohort was applied to all 47 countries in our model for the age categories of under 5 (7.3 % per year), 6 to 20 (1.4 % per year), and 21 to 30 (1.8 % per year) [10]. Beyond 30 years of age, we applied a multiplier of 4.5 to the country-specific probability of mortality in the general population [15] to reflect for the higher mortality risk associated with SCD. The multiplier was derived by comparing the mortality risk in the Tanzanian cohort for six 5-year age increments of 0–5, 6–10, 11–15, 16–20, 21–25, and 26–30 years of 31.5 %, 6.8 %, 6.8 %, 6.8 %, 8.7 % and 8.7 % [10], to those in the general population in Tanzania of 5.4 %, 1.6 %, 1.3 %, 1.6 %, 2.1 %, and 2.9 % [15], and taking average ratio of these six age-pairs. Finally, a disability weight of 0.04 was used to account for the disability burden associated with the presence of SCD, based on an assessment of the impact of anemia without painful crises or other complications [16].

### Cost inputs

All costs were expressed in 2014 US$. The cost per IEF was assumed to consist of the cost of equipment, materials and the labor cost to perform the analysis. We assumed all isoelectric focusing would be conducted using the GE Healthcare Multiphor II Electrophoresis system # 18-1018-06, which is available at a cost of $4,210 per unit [17]. The fixed cost was converted into an average cost per screening on the basis of a useful life of 5 years and a maximum number of screenings per day of 80, e.g. 19,200 per year at full capacity. The daily limit of 80 was chosen, because each machine can run up to 80 samples simultaneously. This translates into an average cost per sample of US$ 0.044 at full utilization. We assumed that the quantity of IEF systems must be represented by a whole number and countries with fewer than 19,200 live births per year would still need to purchase one analyzer with the fixed cost of the analyzer spread out over fewer tests.

The variable cost per screening was estimated at $9.90 per test, which was based on an internal analysis of cost data at the Uganda National Health Laboratories, Ministry of Health Kampala, Uganda. This cost estimate accounts for the reagents used in the testing process ($1.80 per sample), collection materials, which included a dried blood spot kit, gloves, cotton and water ($1.60 per sample), the cost of overhead, which included professional salaries as well as the rent of the testing facility ($5.00 per sample) and the transport of the sample to the centralized testing facility in Kampala, Uganda by means of the postal system as well as the communication of test results back to the local testing facilities ($1.50 per sample). Due to the possibility of sample contamination, this process would be repeated in the case of a positive screening result.

Newborns with a positive confirmatory IEF result would be initiated on interventions to reduce the mortality risk from malaria and other infections. The interventions include pneumococcal vaccination during the first year of life using a three-injection regimen at a cost of US$ 26.88 per injection (US$ 80.64 total) based on local pharmacy costs obtained from Aminu Kano Teaching Hospital, Kano, Nigeria. For malaria prevention, an insecticide-treated mosquito bed net would be provided and assumed to be replaced annually at a unit cost of US$ 10.00 [18], lifetime prophylactic antimalarial therapy with proguanil 100 mg at a cost of US$ 0.0479 per day (US$ 17.48 per year) for children age 0–15 years and proguanil 200 mg at a cost of US$ 0.0958 per day (US$ 34.97 per year) for young adults age 16 and older [19], lifetime supplementation with folic acid 5 mg at a cost of US$ 0.0026 per day (US$ 0.95 per year) [19], prophylactic penicillin V 125 mg (5 ml) twice daily at a cost of US$ 0.082 per day (US$ 29.93 per year) for children age 0–5 and prophylactic penicillin V 250 mg (5 ml) twice daily at a cost of US$ 0.138 per day (US$ 50.37 per year) for children age 5–10 [19].

### Scenario and sensitivity analyses

To test the robustness of model results, select inputs were varied in one-way sensitivity analyses for countries where newborn screening for SCD was found to be cost-effective at baseline. In addition, the incidence estimates for the countries included in our model may have been at least partially based on childhood or adult testing, not necessarily newborn screening. As a result, the true incidence rate may be higher than reported. We therefore tested the impact of a doubling in the incidence rate in scenario analysis #1. In the same spirit, it is possible that the SCD related mortality rate may also be underreported and we have assessed the impact of a doubling in the mortality rate in scenario analyses #2. Finally, probabilistic sensitivity analyses (PSA) were performed by running 10,000 iterations of the model, while randomly selecting the values for 10 key model inputs from a probability distribution that was defined for each of the parameters displayed in Table 1. This process enabled us to estimate the 95 % confidence interval around the 47 base case incremental cost effectiveness ratio (ICER) estimates, and also the probability that NSPI is cost-effective in each country.

## Results

The annual number of infants born with SCD in the 47 countries in SSA is estimated at 228,169. The annual incidence is unevenly distributed, with estimates ranging from 0 cases in Djibouti, Lesotho, and Mauritius, to 38,217 and 85,186 in Democratic Republic of Congo (DR Congo) and Nigeria, respectively.

Mean life expectancy of unscreened newborns in rural and urban areas across SSA was estimated at 1.7 years and 6.7 years, respectively. All country-specific base case model outputs are displayed in Table 3. The life expectancy of screened and treated infants ranged from 24.2 years in the Central African Republic to 32.4 years in Cape Verde (mean 27.0 years). The increase in life expectancy associated with NSPI relative to no treatment translates into 2,414,612 disability-adjusted life years (DALYs) that could be averted across all countries. Importantly, DALY estimates from DR Congo and Nigeria alone account for more than 50 % of DALYs that could be averted in this analysis.

**Table 3:** Model results

Country name	DALYs averted	Total costs	Incremental cost-effectiveness ration (95 % CI)	Probability that screening is highly cost-effective	Scenario 1: double SCD incidence rate	Scenario 2: double mortality rate
Angola	82,167	$14,874,250	$181 ($115–$710)	99.01 %	$137	$212
Benin	49,138	$7,596,778	$154 ($120–$391)	99.53 %	$120	$178
Botswana	51	$451,506	$8,853 ($4 k–$73 k)^b^	16.92 %	$5,273	$12,990
Burkina Faso	34,415	$9,652,203	$280 ($197–$497)	99.53 %	$177	$318
Burundi	10,309	$3,607,194	$350 ($241–$607)^b^	10.78 %	$214	$408
Cameroon	69,142	$12,962,308	$187 ($148–$855)	98.56 %	$139	$219
Cape Verde	196	$116,771	$596 ($363–$2,220)	98.17 %	$342	$723
Central African Republic	9,967	$2,377,372	$239 ($164–$584)	73.16 %	$163	$281
Chad	22,655	$6,738,454	$297 ($199–$601)	99.90 %	$190	$348
Comoros	285	$271,711	$953 ($367–$12,296)^b^	31.22 %	$517	$1,139
Côte d’Ivoire	35,386	$10,169,638	$287 ($190–$925)	98.36 %	$189	$343
Congo-Brazzaville	15,353	$2,674,621	$174 ($108–$683)	98.37 %	$134	$204
DR Congo	414,120	$62,013,889	$150 ($105–$298)	99.25 %	$117	$171
Djibouti	0	$249,184	n/A^a^	0.00 %	n/A^a^	n/A^a^
Equatorial Guinea	4,060	$586,080	$144 ($98–$323)	99.97 %	$115	$165
Eritrea	139	$1,788,594	$12,852 ($3 K–$125 k)^b^	0.00 %	$4,368	$14,435
Ethiopia	2,198	$25,480,760	$11,591 ($3–$113 k)^b^	0.00 %	$4,267	$13,283
Gabon	7,827	$1,175,675	$150 ($103–$989)	95.54 %	$126	$177
Gambia	4,277	$1,021,570	$239 ($160–$770)	93.14 %	$164	$283
Ghana	57,781	$12,272,350	$212 ($144–$635)	99.28 %	$150	$249
Guinea	57,696	$8,528,250	$148 ($100–$320)	99.44 %	$116	$169
Guinea-Bissau	2,021	$786,453	$389 ($238–$1,150)	69.87 %	$239	$466
Kenya	51,539	$18,968,262	$368 ($245–$749)	99.26 %	$225	$435
Lesotho	0	$556,926	n/A^a^	0.00 %	n/A^a^	n/A^a^
Liberia	5,914	$2,037,429	$345 ($219–$1,007	55.39 %	$215	$410
Madagascar	37,917	$9,988,026	$263 ($185–$523)	93.73 %	$172	$307
Malawi	19,335	$8,411,580	$435 ($245–$1,294)^b^	4.89 %	$258	$513
Mali	29,990	$8,394,677	$280 ($190–$616)	98.29 %	$182	$329
Mauritania	4,774	$1,488,728	$312 ($173–$1,228)	95.98 %	$198	$368
Mauritius	0	$159,242	n/A^a^	0.00 %	n/A^a^	n/A^a^
Mozambique	15,895	$9,851,847	$620 ($348–$1,911)^b^	32.57 %	$350	$735
Namibia	308	$583,765	$1,895 ($810–$12,129)	88.26 %	$994	$2,307
Niger	59,276	$12,017,462	$203 ($155–$758)	78.01 %	$141	$234
Nigeria	867,551	$136,436,100	$157 ($118–$630)	99.39 %	$123	$182
Rwanda	6,922	$4,690,792	$678 ($443–$4,102)	11.41 %	$123	$191
Sao Tome and Principe	428	$87,930	$205 ($116–$4,875)	88.64 %	$148	$241
Senegal	28,136	$6,901,261	$245 ($190–$1,091)	97.31 %	$165	$288
Sierra Leone	29,522	$4,580,943	$155 ($111–$622)	98.10 %	$121	$178
Somalia	121	$4,008,936	$33,242 ($21 k–$190 k)^b^	0.00 %	$15,585	$37,835
South Africa	932	$10,364,811	$11,116 ($7 k–$93 k)^b^	2.06 %	$5,354	$13,209
Sudan	51,262	$17,825,373	$348 ($260–$1,603)	97.98 %	$215	$410
Swaziland	37	$341,676	$9,136 ($3 k–$741 k)^b^	1.42 %	$4,729	$11,296
Tanzania	123,313	$28,082,629	$228 ($188–$834)	99.98 %	$155	$265
Togo	23,646	$4,018,804	$170 ($134–$600)	96.11 %	$128	$196
Uganda	114,565	$23,587,765	$206 ($162–$712)	96.65 %	$143	$237
Zambia	59,074	$11,031,575	$187 ($147–$704)	99.71 %	$137	$217
Zimbabwe	4,973	$4,030,324	$810 ($520–$5,827)	16.35 %	$450	$981
Sum/Average	2,414,612	$513,842,475	$213	n/A	n/A	n/A

^a^Not applicable (n/A). The ICER is not defined if the number of DALYs averted in the denominator is equal to zero. For purposes of interpretation, the cost-effectiveness ratios in these three cases are approaching infinity, thus, newborn screening for SCD would not be considered cost-effective

^b^Denotes ICERs that exceed the cost-effectiveness threshold of a cost per DALY averted of less than one time per capita income as per WHO guidelines [11], thus, newborn screening for sickle cell disease would not be considered highly cost-effective

The cost of universal newborn screening using IEF in all 47 countries is estimated at US$312 million per year. Lifetime treatment cost of the prevention package (mean, range) was US$876 (US$828-US$930). The sum cost of a universal NSPI program and the net present value of lifetime treatment costs were US$514 million per year. Most costs would be borne by Nigeria (approximately US$136 million) and DR Congo (approximately US$62 million), but for 34 other countries, the budget impact would still exceed US$1 million per year.

**Fig. 2:**
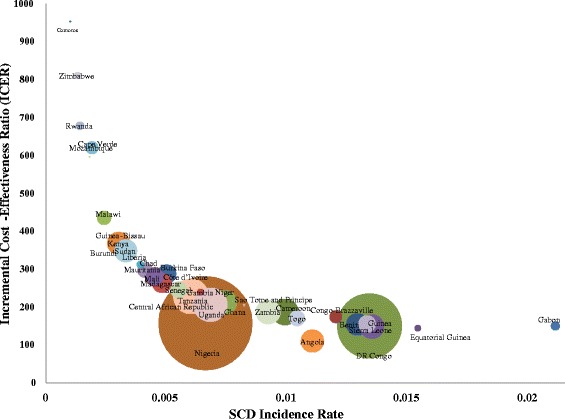
Country ICERs^a^ by incidence rate and DALYs that could be averted^b^. ^a^Only countries are shown where screening for SCD was below US1000/DALY averted. ^b^The circle size represents the number of DALYs that could potentially be averted by routine screening and subsequent treatment of all newborns for SCD

The incremental cost-effectiveness ratios and corresponding 95 % confidence intervals of NSPI in the 47 countries are displayed in Table 3 and, for the subset of 34 countries where screening is cost-effective in the base case, in Fig. 2. The cost per DALY averted ranges from US$144 (95 % CI: $98-$323) in Equatorial Guinea to an ICER approaching infinity in the three countries that report zero burden of SCD and where no DALYs are therefore expected to be averted. The population-weighted average cost per DALY averted for all 47 countries is US$213. Among 34 countries with a cost per DALY averted below one time per capita gross domestic product (GDP), the cost per DALY averted is US$190.

### Results from scenario and sensitivity analyses

Results from our two scenario analyses are displayed in Table 3, columns 6 and 7. The ICER estimates decreased in scenario #1 and increased in scenario #2 relative to basecase results, but the absolute impact of either one of these two inputs was relatively small for most of the countries in our panel. In the case of six countries, Botswana, Burundi, Comoros, Malawi, Mozambique, and South Africa, the ICER exceeded per capita GDP in the basecase, but would fall below this threshold if the incidence rate were to double. Table 4 displays the results from one-way sensitivity analyses. For simplicity, only the high end of the sensitivity range is included and only for countries where the base case estimate indicated that newborn screening for SCD was cost-effective. Generally, the model results proved robust to one-way variations in model parameters. In addition, probabilistic sensitivity analyses suggest that the model results are generally robust to simultaneous variation of all model parameters, as the 95 % CI associated with the ICERs fall below the relevant cost-effectiveness thresholds (Table 3). The probability that the intervention was indeed cost effective with ICER estimates below commonly accepted thresholds generally exceeds 95 % or even 99 %. However, for 10 countries, fewer than 95 % of the iterations fall below the relevant threshold.

**Table 4:** Results of 1-way sensitivity analyses^a^

	SCD incidence-50 %	Discount rate 6 %	Cost of IEF screening $15	Probability survival Urban areas 95 %/year	100 % of population in Urban areas	Probability survival rural areas 75 %/year	IEF specificity 99.9 %	Disability weight of 0.06	IEF sensitivity 99.9 %
Angola	$267	$275	$290	$230	$216	$191	$186	$188	$183
Benin	$228	$225	$193	$210	$193	$165	$159	$161	$156
Burkina Faso	$468	$415	$372	$319	$359	$294	$286	$280	$274
Cameroon	$312	$275	$240	$279	$230	$199	$196	$193	$189
Cape Verde	$1,107	$1,027	$853	$926	$684	$619	$633	$609	$597
Central African Republic	$395	$351	$319	$315	$309	$257	$250	$246	$241
Chad	$519	$444	$410	$405	$341	$323	$312	$305	$299
Côte d’Ivoire	$490	$437	$391	$425	$354	$305	$302	$296	$290
Congo-Brazzaville	$260	$259	$219	$293	$203	$183	$181	$180	$175
DR Congo	$220	$212	$187	$195	$188	$162	$156	$155	$152
Equatorial Guinea	$208	$203	$177	$186	$190	$156	$150	$149	$146
Gabon	$204	$222	$178	$369	$161	$154	$154	$155	$152
Gambia	$392	$369	$317	$367	$284	$251	$250	$245	$240
Ghana	$341	$324	$278	$306	$257	$224	$222	$218	$214
Guinea	$216	$211	$184	$191	$186	$159	$154	$152	$149
Guinea-Bissau	$717	$610	$547	$489	$528	$415	$411	$400	$392
Kenya	$663	$570	$519	$493	$427	$399	$391	$379	$371
Liberia	$605	$544	$477	$479	$421	$364	$363	$353	$346
Madagascar	$447	$404	$357	$321	$339	$282	$277	$269	$264
Mali	$482	$431	$383	$360	$350	$301	$295	$288	$282
Mauritania	$531	$491	$429	$388	$406	$331	$328	$319	$313
Namibia	$3,729	$3,128	$2,825	$2,409	$2,433	$2,034	$2,027	$1,946	$1,908
Niger	$332	$301	$269	$276	$226	$221	$213	$209	$205
Nigeria	$230	$226	$196	$227	$195	$168	$163	$162	$159
Rwanda	$1,279	$1,080	$982	$908	$753	$731	$720	$693	$679
Sao Tome and Principe	$324	$319	$328	$264	$238	$214	$214	$211	$206
Senegal	$484	$380	$330	$306	$324	$261	$258	$252	$247
Sierra Leone	$194	$219	$200	$229	$204	$167	$160	$161	$157
Sudan	$617	$544	$484	$447	$426	$372	$367	$356	$349
Tanzania	$230	$218	$193	$205	$182	$167	$160	$158	$155
Togo	$162	$175	$150	$172	$163	$136	$130	$130	$128
Uganda	$337	$297	$273	$286	$227	$225	$216	$212	$207
Zambia	$293	$271	$242	$241	$246	$201	$196	$193	$189
Zimbabwe	$1,545	$1,287	$1,184	$1,041	$1,054	$869	$863	$832	$816

^a^This table only displays results for those countries where NSPI was cost-effective in the basecase

## Discussion

To our knowledge, this is the first study to systematically evaluate the cost-effectiveness of NSPI programs across most countries in SSA. Based on the commonly accepted threshold of less than one time per capita GDP to assess a highly cost-effective intervention [11], we conclude that NSPI provides excellent value per dollar spent in the settings of many, but not all countries in SSA. We propose to classify countries into three distinct categories: In 24 countries, NSPI is almost certainly cost-effective; in 13 countries, NSPI is either unlikely to be, or is certainly not cost-effective; and in 10 countries, NSPI is probably cost-effective, but the findings are subject to varying degrees of uncertainty. The countries where NSPI is not cost-effective tend to be located in areas with a historically low malaria burden. In Djibouti, Lesotho, and Mauritius, the incidence of SCD is essentially zero. In Botswana, Eritrea, Ethiopia, Somalia, South Africa, and Swaziland, the incidence rate of SCD is approximately 0.01 % or less, or 10 cases out of 100,000 live births, such that the corresponding cost per DALY averted exceeds the World Health Organization (WHO) cost-effectiveness threshold, meaning that significant resources would generate relatively small improvements in population health. However, results from our scenario analysis also suggest that NSPI would be cost-effective in some of these countries if the actual incidence rate in these two populations exceeded that published rates by a factor of 2. In contrast, the results are uncertain in countries with a higher, but still relatively low annual incidence of SCD, around 0.15–0.80 %, or 150–800 cases out of 100,000 live births, such as around Central African Republic, Gambia, Guinea-Bissau, Liberia, Madagascar, Namibia, Niger, Rwanda, Sao Tome and Principe, and Zimbabwe. For these countries, the element of uncertainty is only introduced once multiple parameters are varied simultaneously in probabilistic sensitivity analyses.

In the remaining 24 countries, our model suggests NSPI would provide a very attractive return for investments in healthcare and could lead to meaningful improvements in population health. The average cost per DALY averted for these 24 countries included in the model is US$184, which is not only cost-effective according to the WHO definition, but also compares favorably relative to other healthcare interventions in SSA. For example, the cost-effectiveness of first line antiretroviral therapy for HIV was reported at US$620 per year of life gained in Cote d’Ivoire [20], and is likely to be even higher for second-line antiretroviral therapy where annual treatment costs alone have been estimated at US$1,037 in South Africa [21]. Similarly, the cost-effectiveness of antiretroviral therapy for prevention among serodiscordant couples has been estimated at $590 per life year saved [22]. For vaccines, a review of 44 studies found that the cost per DALY averted was below US$100 in about half of all studies reported and almost a fourth of all vaccine studies reported an estimate exceeding US$500 [23].

Our results support the WHO’s call urging member countries where SCD is a public health problem to establish national health programs, operate specialized centers for SCD and facilitate access to care and treatment including NSPI [24, 25]. Providing access to NSPI in those states will improve childhood survival thereby contributing towards the attainment of Millennium Development Goal 4. While this analysis focuses on currently available interventions for SCD in SSA, in future, technological and medical advances may alter the clinical course of SCD as well as the cost of managing the disease in these countries.

### Limitations

We did not consider a third strategy where infants not screened at birth would be re-evaluated at the onset of symptoms. Screening a symptomatic subset of newborns later in life would reduce the number and cost of screening tests, but given the relatively short life expectancy in this population and the relatively poor access in the region to specialized hematology units to manage complications of SCD, it is not clear whether such an approach would be a viable strategy. However, future research could compare the cost-effectiveness of newborn screening for SSD versus delayed screening at the onset of symptoms. Also, we used mortality estimates from Tanzania across all 47 countries but the natural progression of the disease in the Tanzanian cohort may be different from other settings, especially in countries with a low malaria burden. Furthermore, elements in the prevention package in the Tanzanian cohort differed from those in our cost-assessment, e.g. we included the cost of prophylactic penicillin, which was not the standard of care in Tanzania at the time the study was conducted. In the model, we assumed that patients, or patient caretakers, would comply with the package of prophylactic interventions over their lifetime, however, imperfect compliance would reduce the health economic value of newborn screening. Last, we applied IEF screening costs as observed in Uganda to all countries in the model, but it is possible that these costs may differ in local settings.

## Conclusion

Using IEF to screen all newborns for SCD plus administration of prophylactic interventions to affected children is cost-effective in the majority of countries in SSA.

## References

[1] Fleming AF, Storey J, Molineaux L, Iroko EA, Attai ED (1979). Abnormal haemoglobins in the Sudan savanna of Nigeria. I. Prevalence of haemoglobins and relationships between sickle cell trait, malaria and survival. Ann Trop Med Parasitol.

[2] Makani J, Komba AN, Cox SE, Oruo J, Mwamtemi K, Kitundu J (2010). Malaria in patients with sickle cell anemia: burden, risk factors, and outcome at the outpatient clinic and during hospitalization. Blood.

[3] Scott JA, Berkley JA, Mwangi I, Ochola L, Uyoga S, Macharia A (2011). Relation between falciparum malaria and bacteraemia in Kenyan children: a population-based, case–control study and a longitudinal study. Lancet.

[4] Piel FB, Patil AP, Howes RE, Nyangiri OA, Gething PW, Dewi M (2013). Global epidemiology of sickle haemoglobin in neonates: a contemporary geostatistical model-based map and population estimates. Lancet.

[5] Grosse SD, Odame I, Atrash HK, Amendah DD, Piel FB, Williams TN (2011). Sickle cell disease in Africa: a neglected cause of early childhood mortality. Am J Prev Med.

[6] Platt OS, Brambilla DJ, Rosse WF, Milner PF, Castro O, Steinberg MH, Klug PP (1994). Mortality in sickle cell disease. Life expectancy and risk factors for early death. N Engl J Med.

[7] Tsevat J, Wong JB, Pauker SG, Steinberg MH (1991). Neonatal screening for sickle cell disease: a cost-effectiveness analysis. J Pediatr.

[8] Rahimy MC, Gangbo A, Ahouignan G, Alihonou E (2009). Newborn screening for sickle cell disease in the Republic of Benin. J Clin Pathol.

[9] McGann PT, Ferris MG, Ramamurthy U, Santos B, de Oliveira V, Bernardino L, Ware RE (2013). A prospective newborn screening and treatment program for sickle cell anemia in Luanda, Angola. Am J Hematol.

[10] Makani J, Cox SE, Soka D, Komba AN, Oruo J, Mwamtemi H (2011). Mortality in sickle cell anemia in Africa: a prospective cohort study in Tanzania. PLoS One.

[11] World Health Organization (2011). Cost-effectiveness Thresholds. Choosing Interventions that are Cost Effective (WHO-CHOICE).

[12] Population Division of the Department of Economic and Social Affairs of the United Nations. World population prospects: The 2010 revision. 2011; http://esa.un.org/wpp/ Accessed July 7, 2014.

[13] Lorey F, Cunningham G, Shafer F, Lubin B, Vichinsky E (1994). Universal screening for hemoglobinopathies using high-performance liquid chromatography: clinical results of 2.2 million screens. Eur J Hum Genet.

[14] Population Division of the Department of Economic and Social Affairs of the United Nations (2012). World Urbanization Prospects: The 2011 Revision.

[15] World Health Organization (2011). Global Health Observatory.

[16] Weatherall D, Akinyanju O, Fucharoen S, Olivieri N, Musgrove P. Inherited Disorders of Hemoglobin. In: Jamison DT, Breman JG, Measham AR, et al, editors. Disease Control Priorities in Developing Countries. 2nd ed. Washington (DC): Oxford University Press; 2006.

[17] Carefordscientific.com (2015) GE Healthcare Multiphor II Electrophoresis system # 18-1018-06. In: http://carefordescientific.com/ge-healthcare-multiphor-ii-electrophoresis-system-18-1018-06-multiphor-ii-system-ea/, editor.

[18] United Nations. ‘Let Us Save Our Children; Let Us Resolve Ourselves, Here in Nigeria, to Do All We Can’ to Stamp Out Malaria Death, Says Secretary-General http://www.un.org/News/Press/docs/2011/sgsm13591.doc.htm 2011. Accessed SG/SM/13591-AFR/2183.

[19] Management Sciences for Health (2013). International Drug Price Indicator Guide. 2012 edition.

[20] Goldie SJ, Yazdanpanah Y, Losina E, Weinstein MC, Anglaret X, Walensky RP (2006). Cost-effectiveness of HIV treatment in resource-poor settings--the case of Cote d’Ivoire. N Engl J Med.

[21] Long L, Fox M, Sanne I, Rosen S (2010). The high cost of second-line antiretroviral therapy for HIV/AIDS in South Africa. AIDS.

[22] Walensky RP, Ross EL, Kumarasamy N (2013). Cost-effectiveness of HIV treatment as prevention in serodiscordant couples. N Engl J Med.

[23] Ozawa S, Mirelman A, Stack ML, Walker DG, Levine OS (2012). Cost-effectiveness and economic benefits of vaccines in low- and middle-income countries: a systematic review. Vaccine.

[24] World Health Organization (2006). Sickle cell anaemia. Fifty-ninth World Health Assembly. A59/9.

[25] World Health Organization. Sickle-Cell Disease: A Strategy for the WHO African Region Malabo, Equatorial Guinea: 2010 World Health Organization Regional Office for Africa, Sixtieth Session; 2010

[26] World Bank. GNI per capita, Atlas method (current US$). Washington DC, USA: World Bank. Available online: http://data.worldbank.org/indicator/NY.GNP.PCAP.CD/countries 2014.

